# Co-Ingestion of Rice Bran Soymilk or Plain Soymilk with White Bread: Effects on the Glycemic and Insulinemic Response

**DOI:** 10.3390/nu10040449

**Published:** 2018-04-04

**Authors:** Stefan Gerardus Camps, Joseph Lim, Atsushi Ishikado, Yumi Inaba, Makoto Suwa, Motonobu Matsumoto, Christiani Jeyakumar Henry

**Affiliations:** 1 Clinical Nutrition Research Centre (CNRC), Singapore Institute for Clinical Sciences (SICS), Agency for Science, Technology and Research (A*STAR) and National University Health System (NUHS), Centre for Translational Medicine, Yong Loo Lin School of Medicine, National University of Singapore, 14 Medical Drive #07-02, Singapore 117599, Singapore; stefan_camps@sics.a-star.edu.sg (S.G.C.); joseph_lim@sics.a-star.edu.sg (J.L.); 2 Health Care R&D, Sunstar Inc., Takatsuki, Osaka 569-1044, Japan; atsushi.ishikado@jp.sunstar.com (A.I.); yumi.inaba@jp.sunstar.com (Y.I.); makoto.suwa@jp.sunstar.com (M.S.); motonobu.matsumoto@jp.sunstar.com (M.M.); 3 Department of Biochemistry, Yong Loo Lin School of Medicine, National University of Singapore, S14 Level 5, Science Drive 2, Singapore 117543, Singapore

**Keywords:** soymilk, soy protein, rice bran, dietary fiber, glycemic response, insulin response, glycemic index

## Abstract

The regular consumption of soy products is associated with inverse incidence of type 2 diabetes, and there has been an increasing interest in the glycemia reducing potential of rice bran and its components. In this study, we investigated whether consuming soymilk with the addition of rice bran (fiber) can reduce the glycemic response of a carbohydrate meal. Seventeen healthy Asian men (BMI: 18.5–29 kg/m^2^) participated in this randomized crossover trial. On four occasions, they consumed white bread (two times) and white bread with two different soymilks differing in protein and rice bran content. Blood samples were taken to measure glucose and insulin response over a period of 3 hours. Taking the glycemic index (GI) value of white bread as a reference value of 100, the GI of white bread when co-ingested with rice bran soymilk (RBS) was 83.1 (±7.7) and sugar-free soymilk (SFS) was 77.5 (±10.1), both were lower than white bread (*p* < 0.05). The insulin response of both soymilk treatments was similar to white bread (*p* > 0.05). The glucose/insulin ratio of RBS and SFS were respectively 43.1 (±6.1) and 60.0 (±17.0) and were lower (*p* < 0.05) than white bread (123.5 ± 21.1) during the first 30 min. In conclusion, co-ingestion of low amounts of soy protein with a carbohydrate meal stimulated early-phase insulin secretion and thereby increased blood glucose clearance effectiveness. Furthermore, rice bran-fortified soymilk reduced the glycemic response similarly to soymilk with a greater dose of soy protein. Rice bran and its components offer therapeutic potential for glycemic and insulinemic control.

## 1. Introduction

The prevalence of type 2 diabetes is increasing worldwide, and good glycemic control is crucial for its prevention and management. Dietary interventions aimed at reducing blood glucose, insulin levels, and their fluctuations can be an economic and effective strategy [1]. The glycemic index (GI) is a classification of the blood glucose-raising potential of carbohydrate foods, and there is substantial evidence suggesting that the consumption of low GI foods decreases post-prandial blood glucose and its insulin levels and fluctuations [2,3,4,5,6,7,8]. In recent years, studies have shown that adding fat, fiber, and plant and animal protein to carbohydrate meals can lower the glycemic response [9,10,11,12]. 

Dietary interventions can be helpful to modify the Asian diet, which is generally based on white rice, is carbohydrate-rich, and high GI [13]. Moreover, Asians have been shown to be more susceptible to diabetes than Caucasians and also have a more rapid and severe transition from prediabetes to diabetes [14,15].

Soybean is a rich source of protein, fiber, vitamins, minerals, fat, isoflavones, and phytoestrogens, and the consumption of soy products has been associated with a range of health benefits, such as reducing blood cholesterol levels [16,17], triglyceride levels, blood pressure, and abdominal obesity and was estimated to reduce coronary heart disease risk [18,19,20]. Additionally, consumption of soy products is associated with inverse incidence of type 2 diabetes [21,22,23]. In short-term studies, the consumption of soy products was associated with a reduction in glycated hemoglobin (HbA1c), fasting blood glucose, and insulin [17,18]. Moreover, acute studies have shown that soy products can attenuate the glycemic response [10,24]. This can be explained by the insulinotropic effect that the consumption of protein and amino acids from different sources has shown [10,11,24]. These studies consistently showed that soy products can be used as one of the ingredients in managing glycemic control.

Besides anti-oxidant, anti-inflammatory, and hypocholesterolemic qualities, there has been an increasing interest in the glycemia reducing potential of rice bran and its components [25]. Rice bran is a byproduct of the rice processing industry and is rich in protein, dietary fiber, and lipids. It also contains significant quantities of mineral elements like potassium, magnesium, zinc, and calcium and phytochemicals like oryzanol [26].

In a previous study, our group demonstrated that consuming 11.8 g of soy protein, derived from a sucrose sweetened soymilk, reduced the glycemic response by 25% and showed an increased insulin response. However, this does not reflect the regular serving size of soymilk nor the recommended serving as mentioned by manufacturers. In this study, we investigated whether consuming a regular volume of soymilk (195 mL) with smaller amounts of soy protein but with the addition of rice bran (fiber) can similarly reduce the glycemic response of a carbohydrate meal. It is hypothesized that soy protein and rice bran can synergistically reduce the glycemic response without increasing insulin.

## 2. Materials and Methods

### 2.1. Subjects

The inclusion criteria for participants were healthy, young, Chinese males aged between 21–60 years, who were non-smokers with a body mass index (BMI) between 18.5 to 29 kg/m^2^ and normal blood pressure (<140/90 mmHg). Exclusion criteria were metabolic diseases (such as diabetes, hypertension, etc.), known glucose-6-phosphate dehydrogenase deficiency (G6PD deficiency), medical conditions and/or taking medications known to affect glycemia (glucocorticoids, thyroid hormones, or thiazide diuretics), intolerances or allergies to foods, partaking in sports at the competitive and/or endurance levels, intentionally restricting food intake, and having a fasting blood glucose of more than 7 mmol/L. A total of 18 participants were screened, and 17 participants were recruited. One participant failed screening due to BMI not being within the range (underweight). The study was conducted in accordance with the guidelines laid down in the Declaration of Helsinki, and all procedures involving human participants were approved by the Domain Specific Review Board (DSRB) of the National Healthcare Group, Singapore. This trial was registered as NCT 03447080 (Clinicaltrials.gov). The protocol was well-explained to the subjects, and they gave their informed consent before participation.

### 2.2. Study Protocol

The study was conducted using a randomized, crossover, single-blind design. The participants arrived at the center at between 8:30 a.m. to 9:00 a.m. following a 10–12-h, overnight fast. They were advised not to take part in any strenuous physical activities and not to consume alcoholic beverages the day before the test sessions. Following a 10-min rest, two finger-prick blood samples were collected five min apart to measure baseline blood glucose concentrations. An indwelling catheter was inserted into the antecubital fossa, and a fasting venous blood sample (3 mL) was also obtained via the cannula. The cannula was kept patent by flushing approximately 3 mL of saline into the catheter after every time the blood was drawn. Subsequently, they were given the test food with the instruction to consume it within 15 min. Following the meal, further venous blood samples (3 mL) and capillary blood samples (finger prick) were collected at 15-min interval for the first 90 min (0, 15, 30, 45, 60, 75, and 90 min) and every half hour thereafter (120, 150, and 180 min).

### 2.3. Glycemic Index Methodology

The methodology used to measure the GI is adapted from the Food and Agriculture Organization/World Health Organization (FAO/WHO) guidelines [27]. The blood glucose concentration was measured using finger-prick blood samples. The finger was disinfected using a sterile wipe and punctured using a single-use lancing device. To minimize plasma dilution, fingertips were gently massaged starting from the base of the hand moving towards the tips. The first two drops of blood were discarded, and the next drop (5 μL) was used for testing. The blood glucose concentration in the sample was measured using the HemoCue^®^ 201RT Glucose analyzer (HemoCue Ltd., Dronfield, UK). Quality control assessment based on 8 measurements of quality control solution with the HemoCue 201RT had a coefficient of variation of 0.9% (mean ± standard deviation (SD) of 5.90 ± 0.05 mmol/L)

### 2.4. Insulinemic Index Methodology

For blood insulin analysis, venous blood was collected in 3 mL K_2_EDTA tube. The blood sample was centrifuged at 1500× *g* for 10 min, and the supernatant plasma was pipetted into Eppendorf tubes and stored in a freezer at −80 °C until analysis. Insulin concentration in the plasma samples (μU/mL) was determined using the Cobas e411 immunochemistry analyzer (Roche Diagnostics, Risch-Rotkreuz, Switzerland). Quality control assessment based on 21 measurements of human serum with the Cobas e411 had a coefficient of variation of 2.6% (mean ± SD of 6.36 ± 0.163 μU/mL).

### 2.5. Treatment Meals

In each test session, subjects consumed one of the following foods:

- white bread (control) as a reference
- rice bran soymilk (RBS) (Sunstar group, Osaka, Japan) co-ingested with white bread
- sugar-free soymilk (SFS) (F&N, Singapore, Singapore) co-ingested with white bread

The control meal was performed in duplicate, and all foods were given in portions containing 50–56 g of available carbohydrates. The nutrient composition and contribution can be found in Table 1. A detailed breakdown of the micronutrients composition of RBS and SFS can be found in the Table A1 in Appendix A.

### 2.6. Statistical Analyses

Seventeen subjects completed the study and two subjects were eliminated from the data analysis because one subject was a taxi driver who had irregular sleep hours and the other participated in a school orientation camp, which involved strenuous physical activity. A total of 15 subjects were analyzed.

The glycemic and insulinemic response were expressed as the incremental area under the curve (iAUC) over a period of 120 min by applying the trapezoidal rule and ignoring the values below baseline [28]. The mean values of the two duplicate control sessions were obtained to calculate the iAUC of white bread. The difference in iAUCs between treatment meals were analyzed using repeated-measures ANOVA with post hoc analysis using Fisher’s least significant difference (LSD) test without correction for family wise error [29,30]. Using the iAUC values, the GI of the test foods was calculated using the following formula [27]:

(1) $$GI=\;\frac{iAUC\;for\;the\;test\;food}{Average\;iAUC\;for\;the\;reference\;food}\;\times 100,$$

A similar methodology was adapted in calculating the insulinemic index (II):

(2) $$II=\;\frac{iAUC\;for\;the\;test\;food}{Average\;iAUC\;for\;the\;reference\;food}\;\times \;100,$$

The ratio of the glycemic response over 30 min to the insulinemic response over 30 min by (glucose/insulin) was calculated as follows:

(3) $$Glucose/Insulin=\;\frac{iAUC\;Glucose}{iAUC\;Insulin}\;\times \;1000,$$

Values are reported as means ± standard error of means (SEM). Statistical analyses were performed using SPSS version 23.0 (IBM Corp, Armonk, NY, USA), and statistical significance was set at *p* < 0.05.

## 3. Results

The baseline subject characteristics are shown in Table 2.

**Figure 1:**
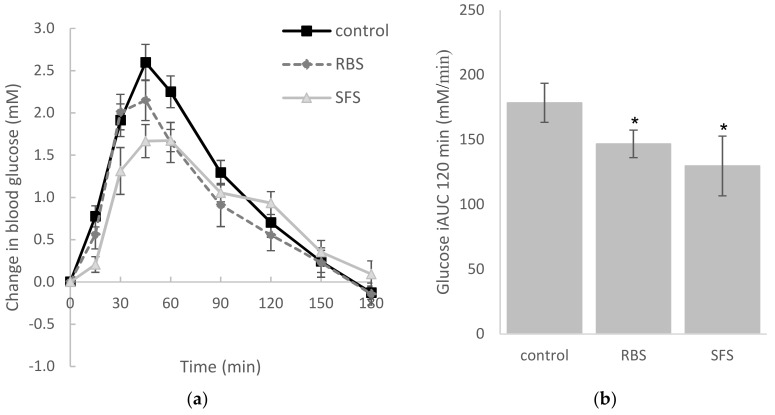
Change in blood glucose over 180 min (**a**) and incremental area under the curve (iAUC) over 120 min (**b**) for the reference and test foods. * (*p* < 0.05) different from the control. RBS: rice bran soymilk, SFS: sugar-free soymilk.

Taking the GI of the white bread control as a reference value of 100, then the GI of white bread when co-ingested with RBS was 83.1 (±7.7) (*p* < 0.05), and the GI of white bread when co-ingested with SFS was 77.5 (±10.1) (*p* < 0.05). Both of the soymilk treatments resulted in a significantly lower glycemic response compared to the control (*p* < 0.05), but there was no difference in the glycemic response between the soymilk treatments (*p* > 0.05) (Figure 1).

**Figure 2:**
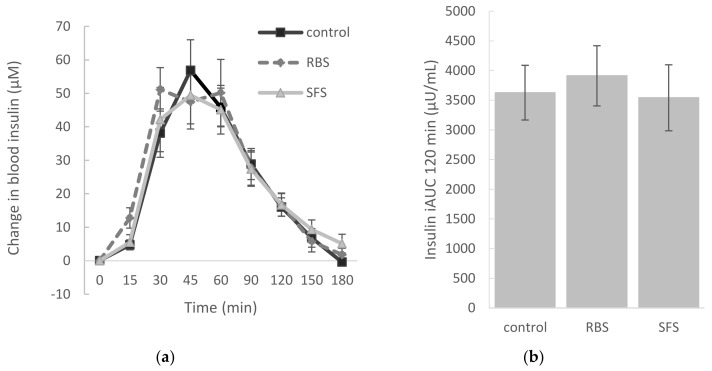
Change in insulin over 180 min (**a**) and incremental area under the curve (iAUC) over 120 min (**b**) for the reference and test foods. * (*p* < 0.05) different from the control. RBS: rice bran soymilk, SFS: sugar-free soymilk.

**Figure 3:**
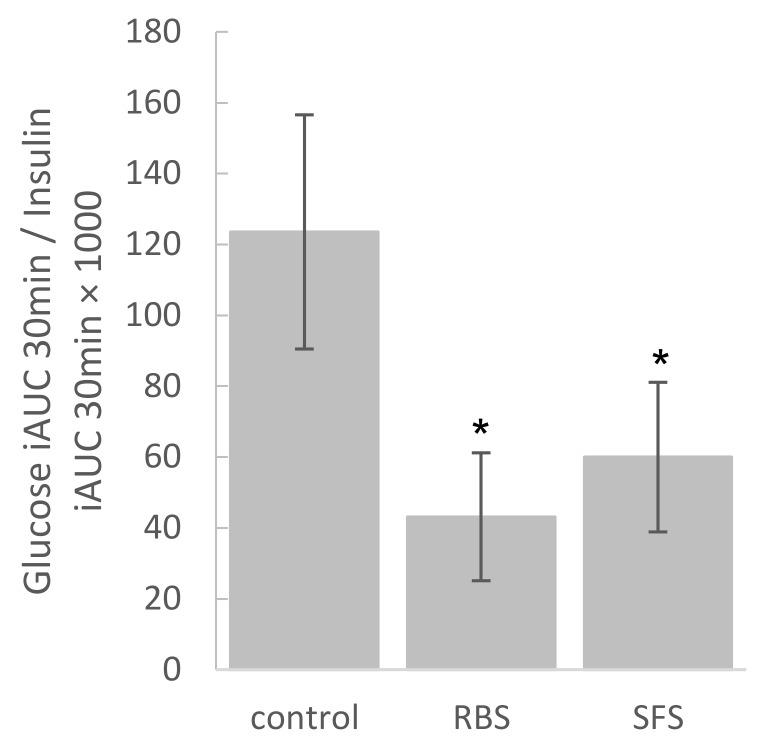
Ratio glucose/insulin during first 30 min for the reference and test foods. * (*p* < 0.05) different from the control. RBS: rice bran soymilk, SFS: sugar-free soymilk.

The insulin response over 120 min of both the RBS and SFS treatments were similar to the control (*p* > 0.05). Taking the II of the white bread control as a reference value of 100, then the II of white bread when co-ingested with RBS was 111.4 (±12.0) (*p* > 0.05), and the II of white bread when co-ingested with SFS was 99.0 (±9.5) (*p* > 0.05) (Figure 2). However, the glucose/insulin ratio over the first 30 min after RBS and SFS consumption were respectively 43.1 (±6.1) and 60.0 (±17.0) and were significantly lower (*p* < 0.05) than the control (123.5 ± 21.1) (Figure 3). This implies that after the consumption of white bread with soymilk, there was a significantly higher release of insulin during the first 30 min relative to the influx of blood glucose when compared with white bread consumption alone. The main study findings were not altered when up to 180 min of glucose and insulin data were included.

## 4. Discussion

In a series of studies that contained plant proteins dispensed as beverages, it has repeatedly been demonstrated that these proteins are insulinotropic and are therefore effective in reducing the glycemic response of foods [10,11,24]. The average amount of proteins in these beverages has been in the region of 12–24 g. In our present study, we have demonstrated that compared to 7.8 g of protein, a considerable reduction in the soy protein content (3.4 g) of a beverage but the inclusion of rice bran can elicit a similar reduction in the glycemic response.

In our previous study, it was demonstrated that co-ingestion of soymilk reduced the glycemic response of white bread by about 25% [24]. The current study demonstrated that consuming soy protein derived from soymilk at lower doses (rice bran soymilk: 3.4 g, sugar-free soymilk: 7.8 g), when compared to our previous study (11.8 g), is still capable of lowering the glycemic response by about 17% (RBS) and 23% (SFS). This finding aligns with our hypothesis that co-ingestion of soymilk with a carbohydrate meal reduces the glycemic response, and this can be a potential dietary strategy to improve glycemic control.

Dietary protein has been shown to have insulin stimulating effects [31,32,33]. In a mouse model study, it was demonstrated that the presence of amino acids and insulin triggered a greater uptake of glucose into muscle tissue than insulin alone [34,35]. This suggests that circulating amino acids in the body can promote the uptake of glucose by stimulating insulin secretion. Amino acids have been shown to stimulate gut peptides like glucagon-like peptide-1 (GLP-1), cholecystokinin (CCK) and peptide tyrosine tyrosine (PYY), and GLP-1, in particular, can increase early-phase insulin secretion [36,37]. Increased insulin has been found by our lab after ingestion of soymilk and amino acids from chicken essence [10,24,38]. In contrast, in this study, no increased insulin response was observed over two hours after the consumption of the soymilks as compared to the control. However, the result did show an increased level of early-phase insulin secretion relative to the influx of blood glucose after the co-ingestion of bread with soymilk. These results indicate that lower doses of protein are still capable of increasing glucose uptake, probably because of the increase in early-phase insulin secretion. The loss of early-phase insulin secretion is a symptom of type 2 diabetes and glucose intolerance [39,40,41] and is associated with hyperglycemia [42]. This highlights the importance of early insulin secretion in glucose metabolism (i.e., given the same amount of insulin exposure, early exposure to insulin effectively clears blood glucose compared to delayed exposure) [39].

Our results showed a similar glycemic and insulinemic response between soymilk with 7.8 g of protein and soymilk with 3.4 g of protein but fortified with rice bran. It is likely that the addition of rice bran to soymilk leads to a similar decrease in the glycemic response even at a lower dose of protein. This is consistent with previous studies that have shown the glycemia-reducing potential of rice bran [43,44]. Besides fiber and protein, other components of rice bran, such as oryzanol, potassium, magnesium, zinc, and calcium, have been shown to have protective properties against diabetes. The phytochemical oryzanol has seen increased interest as one of the active components in rice bran that improves the glycemic response. Recently, Jung et al. showed increased glucose uptake in insulin-resistant cells in vitro [45]. Rice bran is also rich in potassium, and diets low in potassium have been associated with an increased risk of diabetes in epidemiological studies [46]. Magnesium, zinc, and calcium supplementation has been shown to improve glycemic control, and cellular pathways involving these minerals have been linked to glucose and insulin homeostasis [47,48,49,50].

Another possible mechanism by which soy protein and rice bran can help to reduce the glycemic response may include delayed gastric emptying, as demonstrated previously [24]. Reduced gastric emptying slows down glucose release into the intestine and therefore reduces glucose absorption into the systemic circulation and results in a reduced glycemic response.

This study excluded female participants to avoid the well-known metabolic variability in insulin sensitivity and the glycemic response due to the menstrual cycle, which might bias the results due to the gender of the subjects [51]. Given the small carbohydrate contribution by soymilk, there is a minor possibility that the replacement of white bread starch with available carbohydrates from soymilk causes the reduction in the glycemic response. However as previously discussed, the literature supports the hypothesis that protein and fiber help reduce glycemia.

## 5. Conclusions

Co-ingestion of low amounts of soy protein with a carbohydrate meal stimulated early-phase insulin secretion and thereby increased blood glucose clearance effectiveness. Furthermore, rice bran-fortified soymilk reduced the glycemic response similarly to soymilk with a greater dose of soy protein. Rice bran and its components offer therapeutic potential for glycemic and insulinemic control. Co-ingestion of rice bran soymilk with carbohydrate meals can be a simple dietary strategy to improve glycemic control and help in the prevention and management of diabetes.

## Figures and Tables

**Table 1:** Nutrient composition and portion size served.

Meal Characteristic	Control	RBS	SFS
Volume of liquid (mL)	195	195	195
Amount of bread (g)	95.8	89.5	91.4
Calorie (kcal)	251.9	331.3	312.5
Protein (g)	9.5	12.3	16.9
Total CHO (g)	52.4	65.0	54.8
Dietary fiber (g)	2.4	8.6	4.8
Available CHO (g)	50.0	56.4	50.0
Fat (g)	1.8	5.1	5.3

RBS: rice bran soymilk, SFS: sugar free soymilk, CHO: carbohydrate.

**Table 2:** Anthropometric characteristics of the study participants (*n* = 15).

Characteristic (*n* = 15)	Mean ± SEM	Range (min–max)
Age (years)	28.6 ± 1.6	22.4–41.6
Height (cm)	174.1 ± 2.1	158.0–189.0
Weight (kg)	68.3 ± 2.3	52.8–82.8
BMI (kg/m^2^)	22.5 ± 0.6	19.4–26.5
Systolic blood pressure (mmHg)	120.1 ± 2.3	106–139
Diastolic blood pressure (mmHg)	71.6 ± 2.5	59–90
Waist circumference (cm)	79.4 ± 1.8	72–93
Hip circumference (cm)	96.5 ± 1.6	85–105
Fasting blood glucose (mmol/L)	4.7 ± 0.1	4.0–5.4

BMI: body mass index, SEM: standard error of means.

## Appendix A

**Table A1:** Macro- and micro-nutrient composition of rice bran soymilk (*ρ* = 104/100 g/mL ) and sugar-free soymilk per 195 mL.

Macro- and Micro-nutrient	RBS	SFS
Energy (kcal)	96.0	72.2
Protein (g)	3.4	7.8
Fat (g)	3.4	3.5
Total carbohydrate (g)	16.1	4.8
Available carbohydrate (g)	9.7	2.3
Dietary fiber (g)	6.4	2.5
Sodium (mg)	8–25	5.9
Iron (mg)	1.3	-
Potassium (mg)	265	-
Magnesium (mg)	116	-
Calcium (mg)	12.5	390
Vitamin B1 (mg)	0.20	-
Vitamin E (mg)	1.0	-
Vitamin K (mg)	0–8	-
Niacin (mg)	5	-
Vitamin B6 (mg)	0.4	-
γ-Aminobutyric acid (GABA) (mg)	3	-
Oryzanol (mg)	23.8	-
Sucrose (g)	1.0	-

RBS: rice bran soymilk, SFS: sugar free soymilk.
